# A qualitative study of referral to community mental health teams in the UK: exploring the rhetoric and the reality

**DOI:** 10.1186/1472-6963-7-117

**Published:** 2007-07-25

**Authors:** Carolyn Chew-Graham, Mike Slade, Carolyn Montana, Mairi Stewart, Linda Gask

**Affiliations:** 1 School of Community Based Medicine, University of Manchester, Rusholme Academic Unit, almer Street, Manchester, M14 5NP, UK; 2 Health Services Research Department (Box P029), Institute of Psychiatry, King's College, London, SE5 8AF, UK

## Abstract

**Background:**

Generic community mental health teams (CMHTs) currently deliver specialist mental health care in England. Policy dictates that CMHTs focus on those patients with greatest need but it has proved difficult to establish consistent referral criteria. The aim of this study was to explore the referral process from the perspectives of both the referrers and the CMHTs.

**Methods:**

Qualitative study nested in a randomised controlled trial. Interviews with general practitioner (GP) referrers, CMHT Consultant Psychiatrists and team leaders. Taping of referral allocation meetings.

**Results:**

There was a superficial agreement between the referrers and the referred to on the function of the CMHT, but how this was operationalised in practice resulted in a lack of clarity over the referral process, with tensions apparent between the views of the referrers (GPs) and the CMHT team leaders, and between team members. The process of decision-making within the team was inconsistent with little discussion of, or reflection on, the needs of the referred patient.

**Conclusion:**

CMHTs describe struggling to deal with GPs who are perceived as having variable expertise in managing patients with mental health problems. CMHT rhetoric about defined referral criteria is interpreted flexibly with CMHT managers and Psychiatrists concentrating on their own capacity, roles and responsibilities with limited consideration of the primary care perspective or the needs of the referred patient.

**Trial Registration number:**

ISRCTN86197914

## Background

The direction of mental health policy in the United Kingdom (UK) over the last two decades has had two main thrusts: to prioritise the "severely mentally ill" and to move away from a traditional hospital-based and institutional approach towards community-based services which focus on the needs of the individual[1]. At the same time, National Health Service (NHS) policy has stressed the importance of a 'primary care led NHS' [2]. Generic Community Mental Health Teams (CMHTs) are now the main vehicle for co-ordinating and delivering specialist community mental health care in England [3-5]. The concept of the multidisciplinary CMHT, usually consisting of community psychiatric nurses and social workers with input from a clinical psychologist, occupational therapist and psychiatrist, as the focal point of the interface between primary care and specialist mental health care has evolved in the UK over the last quarter century [6]. General Practitioners (GPs) have no other referral options for patients with severe and enduring mental health problems. The Department of Health has consistently recommended that CMHTs should refine their role and focus care only on those patients with greatest need [3,7-9]. It has, however, proved difficult to establish consistent priorities due to the difficulty of defining "greatest need" and because of a lack of alternative provision for patients with common mental health problems. The NICE guidelines suggest a stepped care approach to the management of mental health problems [10] which *should *make it easier to clarify patient need and manage the primary-secondary interface.

The four-fold increase in levels of referral between 1971 and 1997 in the Netherlands, where the system is similar to that in the UK [11], indicates that effectively managing the primary-secondary care interface should be a priority. A key issue for CMHTs, therefore, is how they gate-keep access to their service [12]. Previous studies have suggested that gate-keeping decisions have been largely determined by individual clinicians and teams, rather than through formal strategic control [13]. Wells [14] described how the lack of prescriptive guidelines and under-resourcing at a local level, combined with a professional imperative to demonstrate the meeting of users' needs, was countered by the actions of individual practitioners reshaping users' perceptions of their needs to match available resources. In addition, the tension is resolved and policy objectives met through individual policy interpretation by practitioners. King [15] also described the CMHT members' perceptions of their role, outlining their tension between the team's needs to manage referral pressure, comply with strategic demands and its members' clinical experience and valued practice.

The aim of this paper is to explore the function of the CMHT in managing referral decisions at the primary-specialist interface from the perspectives of *both referrers *(general practitioners, GPs) *and referred-to *(CMHT team leaders and Psychiatrists).

## Methods

The qualitative work reported in this paper formed part of a multi-site cluster randomised controlled trial (ISRCTN86197914) to investigate the use of the Threshold Assessment Grid (TAG) [16,17] to improve access to adult mental health services. The trial investigated the impact on appropriateness of referral of asking GPs in the intervention group to include a completed TAG schedule when referring patients to adult CMHT, in addition to their normal referral practice [18].

The study was approved by Metropolitan Multi-centre Ethics Committee (04/MRE11/8) with Local REC approval in London and Manchester, and research governance support from the relevant Primary Care Trusts and Mental Health Trusts.

### Design

A nested qualitative study utilising two data sources: semi-structured interviews with GP referrers and CMHT team leaders and Consultant Psychiatrists and recordings of CMHT 'allocation' meetings at which decisions were made about whether new referrals should be accepted and the patient assessed by a member the CMHT. Ethical, local Research & Development (R&D) and primary care R&D approvals were obtained for each site.

### Setting

Croydon, South London and Manchester, United Kingdom. The practices and CMHTs in these towns serve inner city and urban populations, with some pockets of deprivation, residents from many ethnic groups (including Black-Caribbean and South Asian, Eastern European), refugees, many areas of high unemployment and poor housing stock.

### Participants

Practices in the trial were randomly allocated to either intervention or control groups, GPs from both groups were invited to participate in the nested qualitative study. Purposive sampling of GPs was used to ensure variation in GP gender, ethnicity and experience and representation of GPs from practices varying in list size. Thirty-five interviews with GPs were carried out (13 control, 21 intervention).

All team leaders (12) and Psychiatrists (14) in the TAG study were invited to participate in interviews and a total of 17 (12 team leaders and 5 psychiatrists) consented and were interviewed.

More detail of the trial CMHT and practice participants can be found in the Trial report [18].

### Data collection

The interview schedule was a flexible topic list which guided a dialogue between the interviewer and respondent. All participants were asked about the function of the CMHT, the referral process and the concept of "appropriateness", and the use of TAG. In addition, interviews with GPs explored their relationship and communications with the CMHT, and topics covered in interviews with secondary care participants (CMHT leads and psychiatrists) included team structure and function, decision-making about referrals, perceptions of the role of other team members as well as relationships with GPs.

A few interviews with GPs were carried out at the beginning of the trial, with further interviews being carried out through-out and at the end of the trial. The themes emerging from analysis of the GP data directed the interview schedules with CMHT leaders and Psychiatrists, which were carried out towards the end of the trial in order to explore particularly how the use of TAG had impacted on their work.

In addition to the semi-structured interviews, all the CMHT members in the teams in the trial were asked to give consent for taping of their routine 'allocation' meetings at which new referrals were discussed, both at the beginning and end of the study. 10 pre-study meetings were recorded (Croydon = 7, Manchester = 3) and 7 post-study (Croydon = 7). Four sets of full meeting (pre and post study) data were achieved.

### Analysis

All interviews were audio-taped with written consent and transcribed verbatim. The interview schedule was modified in the light of emerging data and interviews were continued until category saturation was achieved. In order to ensure that reported data remained completely anonymous, all participants were coded via GP practice or CMHT name (by allocation of numbers) and role.

Analysis was completed independently by four of the authors from the research team with differing professional backgrounds (general practice, psychiatry, nursing and psychology), with themes agreed through discussion. An iterative approach to analysis using constant comparison [19] with the interview schedules for both data sets being modified throughout the study to take into account of themes emerging from the data. Deviant cases and discomfirmatory evidence were actively sought through-out the process of analysis [20].

Content analysis of the transcripts of meetings was carried out and an attempt was made to identify factors which governed decision-making in the allocation meetings.

## Results

The nested qualitative study attempted to explore what factors influenced why referrals were made (from a primary care perspective) and why referrals were accepted or rejected (from the CMHT perspective). The analysis suggested that respondents' own understanding of the purpose and function of the CMHT influenced both referral to and acceptance by the CMHTs. Thus, this paper will explore professionals' views about the functions of the CMHT, explanations of how decisions were made about eligibility for care by the team, and views about the way in which the primary/secondary interface functions. The paper will utilize and present data from the qualitative interviews, and transcripts of the allocation meetings. Data presented in this paper are identified by respondent's profession (*GP *– General Practitioners, *TL *– Community Mental Health Team Leaders and *Psych *– Consultant Psychiatrists) and team or practice identifier.

### Function of the CMHT

All respondents, both GPs and respondents from CMHTs, described the function of CMHTs in general terms, and as defined by current policy documents, to provide care for patients with severe and enduring mental health problems:

'...The function of the team is to assess and treat people with severe and enduring mental health needs'. (TL 24/3)

GPs articulated their need for the CMHT to carry out the assessment of a patient in crisis but also to support them in assessing and managing people that they themselves were having difficulty managing:

'...where you feel you've done everything you can and you're not getting anywhere, so it's for advice as much as anything else.' (GP 643)

'...I wanted a consultant's opinion rather than it being an urgent situation where somebody was suicidal, it was just a consultant's opinion where, where a patient was really, extremely challenging to treat...(GP 601)

'...and sometimes you feel, you know, you're treating the patient and you still need the support. Just to, sort of, for the consultant or specialist to say,"'yes, what you're doing is right, just carry on".'(GP399)

Some GPs suggested that it was a Consultant or Specialist opinion for "expert knowledge", rather than team input, that they needed.

CMHT leads agreed that the team should have the dual roles of carrying out an *assessment *of patients referred to them (often in crisis) and the need to provide some *continuing care *for some patients:

'*well, obviously when there is a crisis, that's an easy referral....' *(TL 5/2)

'...I'd always view the CMHT as having a primary and integral role as the first port of call in terms of accessing the secondary services...' (TL 10/1)

'....providing community input to people with long standing mental health needs and attempting to maintain some kind of optimum level of health at home and prevent hospital readmission and sort of improve the quality of life for people with mental health needs' (TL 23/2)

Psychiatrists' views echoed those of their CMHT team leaders:

'...the ones (patients) who need help in terms of assessment of their mental state or the risk, ongoing risk, or some treatments which can be carried out in the community...' (Psych 24/1)

'I think the CMHT has probably got two principle functions, which is probably the thing that is difficult for us combining the two. I think the bulk of the work that they do is with patients with severe and enduring mental illness where they act as care co-coordinators and provide a range of interventions and support and monitoring functions for some quite poorly people, with a whole range of severe mental illnesses. But then this team in addition to that is providing initial assessment, crisis response, triage type service for referrals from a variety of sources...' (Psych 23/1)

CMHT leads identified a tension in trying to fulfil more than one role:

'...not necessarily always sort of psychosis or and enduring mental illness, but a long term problem that that persons been experiencing which hasn't been able to be dealt with in primary care....and it's more difficult there, I mean, that varies, doesn't it?' (TL 23/1)

'...theoretically, we have an operational policy which suggests we work with people with severe mental health problems, but we get all kinds of people referred...'(CMHT 15/2)

Reflection over this lack of clarity and how they managed it within the team often led CMHT leaders to suggest that their own team did not necessarily behave in the same way as neighbouring teams in order to accommodate more referrals and offer flexibility:

'...I think the way that we work, I suppose we have our own characteristic, which is, I think, we're a very flexible team, we probably have a lower threshold for accepting assessments than others...' (TL 12/1)

Interviews with all the CMHT respondents contain reflections and admissions of the conflicts they work within – conflict between the need to be responsive to crises, and to carry out acute assessments, and their role in providing ongoing care for people with long term (severe and enduring) problems, and some respondents were unhappy with the recent policy directives that governed their roles:

"...we've become too specialised...used to see everything... [I] used to enjoy the variety of having brief interventions...now looking after people for 5–7 years...the service has not grown to look after those we used to see like people with depression, anxiety and neuroses..." (CMHT 23/2)

### Referral- a confusion of purpose?

All GPs interviewed recognized that the role of the CMHT was to provide care for people with severe mental illness, although there were varying views on what constituted "severe mental health problems" and some GPs described a lack of clarity over criteria for referral and noted that there had been a change over time in the sort of patients accepted by the team:

'...You've got to be very fantastically suicidally depressed to reach their criteria actually.' (GP 657)

'...well its also just that they're a bit awkward, I think, they always managed to see a few people without schizophrenia and bipolar, of the most difficult cases, and now they've stopped doing it. I think they've just decided that they're not going to do it...' (GP 652)

Some GPs felt that more clarity about referral criteria was needed, whilst others wanted *less *restriction on their ability to refer:

'...I think it should be left quite open and if a GP feels that they need the CMHT, then that's what should happen rather than having strict criteria then you will have patients on the boundary and where do they go?...' (GP 273)

GPs generally viewed CMHTs as trying to avoid taking on referrals and describe a variety of strategies in order to force the desired response from the team:

'if you don't get a tick in the right box you're in real trouble and you have to make an effort and start jumping up and down and swearing at people' (GP 616)

'...but you don't want to say somebody's dangerous when really you don't think they are just to get them seen quicker. I wouldn't do that.' (GP 638)

Some CMHT respondents felt that the role of the GP should be to carry out an adequate risk assessment themselves *prior *to referring the patient to the team, but others suggested that they understood the reasons why some GPs could not do a reliable risk assessment:

'...well, they don't seem to even try to make an assessment, they just refer...some GPs, anyway...' (TL 18/3)

*'...I don't feel that they should have make those decisions*, [about who to refer] *they have enough decisions to make, you know...I don't think they should have to do that...' (TL 23/1)*

'...I think for a lot of GPs it's.... for them, there are issues around how comfortable or competent they feel in dealing with people with mental health issues' (TL 17/8)

*'...they might not have, em, .felt confident in doing that (*risk assessment*), and having to put something to paper which they could be later on taken up on...' (Psych 23/1)*

There was evidence that CMHT leads recognized the tactics used by GPs, particularly describing how some GPs exaggerated the risk posed by the patient in order to ensure that the referral was accepted:

'...GP might be inclined to, on the referral, to exaggerate the risk...they shouldn't have to make those risk assessments, you know, it's then another role for GPs who are very busy and have five or ten minutes with client maximum, makes it difficult..." (TL 23/1.7)

Psychiatrists attached importance to their prior knowledge of the GP making the referral and to the history of previous referrals from that GP:

'..there are few GPs, not ones that I work with, that you tend to know are more anxious, so they'll be more panicky about somebody's level of risk. And... there are other GPs who..... are more relaxed, so, you know they may say somebody who is not risky, and you may feel more, you know, more anxious about that, erm, cos you know, you know some people are much happier carrying a degree of risk than other people...you do get a feel for the GPs and their thresholds for requiring, requesting support.' (Psych 22/1)

'Well we sometimes hear ourselves say "well I'm not sure whether that person is suitable or not but that GP doesn't refer very often and they're normally very astute in their assessment so lets give it a go" would be one thing. Does it work in the opposite direction? Probably without us being too conscious of it. There are certainly some GPs who we perhaps think aren't good referrers and that might make us less inclined to just accept their referrals' (Psych 23/1)

### Psychiatrists- a confusion of role?

As a member of the CMHT, the psychiatrist can influence whether or not referrals are accepted by the team. Both Psychiatrist and CMHT respondents described how they perceived that the expert knowledge lay with the Consultant:

'...the consultant provides I suppose expertise and advice specifically around medication and diagnosis...' (CMHT 13/1)

'...I suppose my role would be to the clients that other members of the team are worried about, or if it's not their field of expertise, for example, a diagnosis is required or err medication issues need to be addressed' (Psych 25/1)

But Psychiatrists had great difficulty defining their own individual role within the 'team':

'I think my role is to maybe try and integrate the more medical assessments with the psychological and the CMHT assessments and perhaps, I do believe that doctors do probably have a more holistic view of the patient, in that we have a different perspective in terms of the longitudinal history of the patient, rather than having a cross sectional view. And while, so we have got sort of, a multi disciplinary view, but also I think a more longitudinal assessment of the patient.' (Psych 23)

Similarly GPs had some difficulty in defining whether the Psychiatrist was part of the team or separate from it:

'...they don't come out into the community, they don't, they have never made that transition, they live behind the brick walls of the hospital...' (GP 628)

'....part of the team as opposed to being the top of the very narrow pyramid...' (GP 643)

CMHT respondents also expressed very different views on the role of the psychiatrist and how they fitted into their team. Some respondents saw the consultant as a leader:

' ...both consultants take a very, very strong lead in team...' (TL 17/2)

'...they have the sort of final say, I would say, within our team in the meetings about whether it's appropriate or not appropriate...' (TL 23/1.2)

or reported that the Psychiatrist behaved as a leader, without being integrated into the team:

'...Our psychiatrist continues to work in the way that he's always worked. And so therefore it's been, the process has been more slow I think for this team in developing in different ways...' (TL 11/7)

'...Occasionally they'll make kind of unilateral decisions that may affect other people, wont always communicate that, they don't always perceive the need to...' (TL 10/3)

Others reported that the Psychiatrist was disengaged from the team:

'...he doesn't see himself as working in the community at all.' (TL 23/1.2)

and more particularly from the GPs:

'...there's a big gap between our consultant and the GPs, you know, he doesn't have any direct contact with them he doesn't know them, so that changes the relationship.' (TL 23/1.5)

However psychiatrists were well aware of the tensions within the team around their own role:

'My main role myself is, I think is from the clinical point of view of being a leader for the whole team...'(Psych 24/1)

Particularly when it came to deciding who should or should not be seen by the team, which sometimes led to disagreement with the rest of the team:

'...And usually it's me then saying 'I think we should see this patient'. I think CPNs, psychologists have more the tendency to say "Well we should really not see this patient".' (Psych 23/1)

### How were decisions about referrals made? The rhetoric and the reality

CMHT leads and Psychiatrists described how decisions were made in their team in a language which reflected current policy rhetoric:

"...(we) advise GPs in particular where we do link-working into other organisations..."(TL 13/1)

Some CMHT leaders described their team as having inclusive criteria in contrast to other services:

*"'..other specialist teams tend to operate quite a strong, what we call it, an exclusive criteria..." (TL 10/2)*.

Others admitted that their service operated very strict criteria for accepting a referral:

"...we offer almost a psychosis service" (TL 24/2)

Attempts to clarify exactly how CMHTs how decisions were made and particularly management issues, indicated that it was sometimes unclear how decisions were made within the 'team':

'*well, how the decision is made? that's a difficult one. how we assess whether it's severe, emm, whether we agree, we don't always, even if there were strict criteria, erm...' (T17/6)*

There was a similarity in the way that the referral system was described at most of the sites:

'...Well it's logged and then the urgent, urgent ones are dealt with on duty, so somebody would go and assess somebody as part of the duty system. And those that are deemed to be routine are brought to referral allocation meeting, and we discuss them. And sometimes we will do things like say this isn't, there's not enough information or you can just tell they're not eligible and we'll make a decision, maybe someone will liase with the GP about that particular client. Or if they are eligible, we allocate somebody to go out and do the assessment. And the next part of the meeting is about feedback from assessments that people have conducted over the past week or so. And then we make a decision about whether they're eligible for services or not'.(TL 24/2)

The *reported process *of decision-making within the allocation meeting also varied little from site to site, although was difficult to tease out in the interviews:

Interviewer: Could you describe your role within the team, please?

*A: That's a big one. How long have you got? (laughter) I mean I see my role at the moment as team leader, as a leader as opposed to team manager. I think there is a difference in that...And my role is to support and kind of lead and direct...... You know empowering, empowering the staff really. So not taking responsibility for them, but giving them the skills I suppose to be able to kind of improve on their role and functioning*.

Interviewer: Okay, excellent. So how does your team work?

A: How does it work?

Interviewer: *Yeah*.

A: I mean we're a team, a multidisciplinary team that's made up of, we have four CPNs (Community Psychiatric Nurses), we have two social workers, we have an occupational therapist, a support worker, medics, a doctor, and SHO (Senior House Officer) and two team secretaries. And very much the team does work together ...it's a team kind of role in accepting referrals and processing referrals. And through that process the team then, you know, takes the information from a referral and will decide you know whether to work with that referral. Whether we need to maybe pass that referral on to other services, and redirect it through, or send it back. But how we decide that, erm... Have I gone off at a tangent? (TL 11/3)

The *taped meetings *demonstrated four areas of discussion about whether or not a referral was accepted (Table 1). Inconsistency was observed in how decisions were made on whether a referral was accepted or not. Conversations tended to switch between clarifying information, inconsequential comments, comments about the referrer, comments about risk and reiterating what has already been said. It was usually difficult to identify a point in the discussion at which a clear decision was made.

**Table 1:** Analysis of the allocation meeting transcripts

**Area of discussion**	**Illustrative data**
Information received about the patient	A: Well what was her problem again originally? A3: There wasn't much in the referral letter was there? CMHT 10 Team Leader: "She's currently under Sure Start for help with her son's behaviour...So it's been turned down by the Mother and Baby Unit....What does anyone think? Seems to me that she's got mild to moderate depression, with no particular risks, and she's only had 20 mg of fluoxetine, so she really should be treated in primary care. " CMHT 11 CPN: It's got "he wants to end it all" (*voice muffled*...) SW: Yeah, it reads as though ... that sounds actually more worrying than the other one... CMHT 23 "Doesn't say. I don't know what's been going on. ....But at the moment she does warrant additional assessment" CMHT 18
Views on referral and referring GP	S1: Not sure I agree with that. It's a very good referral. S2: No, I think it's a good referral, yes. S3: Yes. CMHT 11 "Talk about clogging up the system with people. I mean, he's on 40 mg now." CMHT 18
Capacity of team	CPN: "This woman is having some sort of adjustment, isn't she, difficulties? I'd like to think that we wouldn't have somebody like this in our service for a long period of time. So we need to think, like we've said before, about endings. Short term intervention maybe, couple of appointments and..." CMHT 11
Decisions about management of accepted referrals	CPN: Are we going to see him? Psych: Yes, with the thought of early discharge back to GP. CMHT 16 A1: "It sounds like she's ready to be motivated and if she gets the help in the course of a group there might not be any need for her to have medication." Psych: "I think that sounds appropriate." A1: "What do you want us to do then?" Psych: "Outpatients." CMHT 18

The professional identity of the speaker (eg Psych, CPN)  is given where it could be identified from listening to the recording of the  meeting. Where it was not possible to identify the speaker, a letter and number  indicates different members of the team (and thus diffierent speakers).

The findings from the analysis of the taped meetings were thus in marked contrast to the more structured and organised way in which participants reported that decisions came about within meetings.

## Discussion

### Limitations of the study

Non-consent by many members of the CMHTs to the recording their referral allocation meetings limits the usefulness of the data from transcripts of allocation meetings and may limit the generalisability of this aspect of the study. The number of psychiatrists who agreed to be interviewed was small, limiting the conclusions that can be drawn from the data. Why Psychiatrists were so reluctant to be interviewed was not clear, other than the messages relayed from secretaries back to the research team about them being "too busy" or "not interested in the study". The data were collected at a time of re-organisation of some of the teams, thus the data may also reflect systemic uncertainty.

### Confusion of purpose and role

A lack of clarity exists over operationalisation of the referral criteria, and decision-making about what constitutes an "appropriate" referral, within the CMHTs studied. For the CMHT respondents and Psychiatrists, whose specific role in the team is unclear, the term "severe mental illness" is used with two contrasting meanings. Firstly as a strict medical category serving the function of an effective gate-criterion for prioritising referral access to the team, but secondly as a descriptive term used to guide clinical practice and informed by the teams' broader perception of what constitutes severe and enduring mental health problems (as described by King [15]). This highlights the tension which exists between the service delivery requirement of having to meet a perceived large demand with limited team resources, and fulfil the clinical responsibilities of managing people with complex needs. This tension is echoed in the GP narratives, where there is also confusion over the role and function of the team. GPs wanted a quick response to requests for assessments of patients in crisis, but also the provision of on-going management for a selected few patients. The GPs that we interviewed in this study described confusion over thresholds for referral of a patient and for acceptance of that referral by the team. They reported that they were unable to challenge the criteria because they perceived they were not explicit. CMHT leads, meanwhile, talked about having clearly defined referral criteria (patients with "severe and enduring mental health problems") but their flexible interpretation of these criteria provided, for them, the possibility of having some level of control over their workload. This flexibility of interpretation operated, we observed, as a demand management technique. However, we must also add that this ad-hoc operational flexibility and lack of clarity in decision-making is inconsistent with the trend towards improved clinical governance in CMHTs, which emphasises consistency and transparency of response [21].

Other commentators suggest that a strong adherence to uni-professional values and the absence of a shared philosophy within CMHTs, as well as a mistrust of managerial solutions to the problems of inter-professional working [22] all contributed to limiting the effective function of a CMHT. To this we must add that the specific role expectations of the CMH team members, viewed both internally with respect to the function of the psychiatrist with respect to the team, and externally, in their referral-based interactions with GPs are unclear. There is evidence of role confusion [23], role conflict (the pressure inherent in fulfilling more than one role) and, on the part of some but not all of the psychiatrists, even role-distancing (not being a 'real' member of the team).

Individual teams acknowledged that they worked in different ways to neighbouring CMHTs, which to some extent seemed to depend on the level of integration of the Consultant Psychiatrist into the team. Within the teams, the Psychiatrists saw themselves as "experts" and GPs describe a wish for access to this "expert knowledge" but the team structure (requiring referrals to be made to the team, not the individual psychiatrist) prevented this. The psychiatrist is viewed as an important 'knowledge' resource within the CMHT and how this knowledge is most appropriately utilised should be explicitly considered within teams.

### Decision making: not consistent with the rhetoric

CMHT respondents describe difficulties in managing the decision-making process about referrals at the interface and this was illustrated in the recordings of referral meetings.

CMHTs use pre-existing knowledge of and relationships with the GP to make decisions about referrals to the team, more than the identified needs of the referred patient. Both CMHT leaders and Psychiatrists described how their views on the competence of individual GPs impacted on the decision to accept a referral, and this was seen in all taped meetings. They also expressed strong opinions on what GPs should do before making a referral. Yet inconsistency was evident, as some stated that GPs should do an "assessment", and others expressed no confidence in GPs being able to assess the patient as they would wish (assessment of risk and including the use of the TAG). CMHTs members seemed preoccupied by their own perceived lack of capacity (amounting to role strain), roles and responsibilities (perhaps due to the difficulties described above) rather than considering fully either the nature of the primary care request, or the needs of the referred patient. Indeed there was very little reflection on needs of the individual patient either in interview data or evidence in taped meetings.

Thus the although the rhetoric of the decision making process might be viewed as the stated intention or 'planned behaviour' [24], it was apparent that attitudes and beliefs about the role and capability of primary care, in addition perceptions of the teams ability, capacity and/or specific remit to carry out its advertised intention led to a rather different kind of process actually taking place, from that which was reported to occur.

## Conclusion

The policy of having crisis resolution teams [25] may resolve some of the tension between referral of patients in crisis and the request for assessment or care for patients for whom some additional input is requested, but CMHTs will still have to contend with referrals that they may feel are "inappropriate" with GPs feeling they are at the limit of their capacity to manage an individual patient. It remains to be seen how or indeed whether the expansion in availability of psychological therapies proposed by Layard [26] will impact upon this. What is apparent from this study is that referral criteria will always be open to interpretation, and that this flexibility may serve a purpose on *both *sides of the primary-specialist interface.

## Competing interests

The author(s) declare that they have no competing interests.

## Authors' contributions

CCG contributed to the design of the study, managed the Manchester field work, designed the qualitative study, analysed the data and drafted this paper.

MS was Principal Investigator. He designed the study and the managed the London site. He contributed to the qualitative study design, data analysis, and the writing of this paper.

CM collected data in Manchester, contributed to the analysis and commented on this paper.

MSt collected data in Croydon, contributed to the analysis, and commented on this paper.

LG contributed to the design of the study including the qualitative study, analysis of the data and co-wrote this paper.

## Pre-publication history

The pre-publication history for this paper can be accessed here:


